# Enhancement of ATRA-induced differentiation of neuroblastoma cells with LOX/COX inhibitors: an expression profiling study

**DOI:** 10.1186/1756-9966-29-45

**Published:** 2010-05-11

**Authors:** Petr Chlapek, Martina Redova, Karel Zitterbart, Marketa Hermanova, Jaroslav Sterba, Renata Veselska

**Affiliations:** 1 Laboratory of Tumor Biology and Genetics, Department of Experimental Biology, School of Science, Masaryk University, Kotlarska 2, 611 37 Brno, Czech Republic; 2 Department of Pediatric Oncology, University Hospital Brno and School of Medicine, Masaryk University, Cernopolni 9, 613 00 Brno, Czech Republic; 3 1st Institute of Pathologic Anatomy, St. Anne's University Hospital and School of Medicine, Masaryk University, Pekarska 53, 656 91 Brno, Czech Republic; 4 Institute of Pathology, University Hospital Brno and School of Medicine, Masaryk University, Jihlavska 20, 625 00 Brno, Czech Republic

## Abstract

**Background:**

We performed expression profiling of two neuroblastoma cell lines, SK-N-BE(2) and SH-SY5Y, after combined treatment with all-*trans *retinoic acid (ATRA) and inhibitors of lipoxygenases (LOX) and cyclooxygenases (COX). This study is a continuation of our previous work confirming the possibility of enhancing ATRA-induced cell differentiation in these cell lines by the application of LOX/COX inhibitors and brings more detailed information concerning the mechanisms of the enhancement of ATRA-induced differentiation of neuroblastoma cells.

**Methods:**

Caffeic acid, as an inhibitor of 5-lipoxygenase, and celecoxib, as an inhibitor on cyclooxygenase-2, were used in this study. Expression profiling was performed using Human Cancer Oligo GEArray membranes that cover 440 cancer-related genes.

**Results:**

Cluster analyses of the changes in gene expression showed the concentration-dependent increase in genes known to be involved in the process of retinoid-induced neuronal differentiation, especially in cytoskeleton remodeling. These changes were detected in both cell lines, and they were independent of the type of specific inhibitors, suggesting a common mechanism of ATRA-induced differentiation enhancement. Furthermore, we also found overexpression of some genes in the same cell line (SK-N-BE(2) or SH-SY5Y) after combined treatment with both ATRA and CA, or ATRA and CX. Finally, we also detected that gene expression was changed after treatment with the same inhibitor (CA or CX) in combination with ATRA in both cell lines.

**Conclusions:**

Obtained results confirmed our initial hypothesis of the common mechanism of enhancement in ATRA-induced cell differentiation via inhibition of arachidonic acid metabolic pathway.

## Background

The therapeutic approach based on induced cell differentiation of transformed cells into mature phenotypes is one of the most promising strategies in recent anti-neoplastic treatment. Retinoids represent the most frequently used group of differentiation inducers, both in leukemias and in some types of solid tumors [1-6]. However, evidence of potential toxicity and intrinsic or acquired resistance substantially limits the use of retinoids in clinical protocols.

Special attention has thus been paid to the combined treatment with retinoids and other compounds that are able to enhance or modulate the differentiation effect of retinoids. For example, all-trans retinoic acid (ATRA)-induced cell differentiation in the HL-60 leukemia cell line can be enhanced either by combined treatment with bile acids [7,8] or with inhibitors of the arachidonic acid degradation pathway, especially of lipoxygenases (LOX) and cyclooxygenases (COX) [9-11].

In neuroblastomas, which are the most common extracranial malignant solid tumors of childhood, differentiation therapy with retinoids is of special interest. Because neuroblastomas are classified as embryonal tumors arising from immature cells of the neural crest, the induced differentiation of neuroblastoma cells has become a part of therapeutic protocols [12-16]. In our previous work, we investigated possible ways of modulating the ATRA-induced differentiation of two neuroblastoma cell lines, SK-N-BE(2) and SH-SY5Y, with LOX/COX inhibitors. We used caffeic acid (CA) as an inhibitor of 5-LOX and celecoxib (CX) as an inhibitor of COX-2. Our results clearly confirmed the power of CA to enhance the differentiation potential of ATRA, especially in the SK-N-BE(2) cells, whereas combined treatment with CX led predominantly to the cytotoxic effect [17].

In this study, we focused on a more detailed investigation of the results described above. We performed gene expression profiling of the cell populations treated with the same combinations of ATRA and LOX/COX inhibitors as in our previous experiments, and the results generate new knowledge about possible molecular mechanisms of the enhancement of ATRA-induced differentiation in neuroblastoma cells.

## Methods

### Cell lines and cell cultures

SK-N-BE(2) (ECACC cat. no. 95011815) and SH-SY5Y (ECACC cat. no. 94030304) neuroblastoma cell lines were used for this study. Cell cultures were maintained in DMEM/Ham's F12 medium mixture (1:1) supplemented with 20% fetal calf serum, 1% non-essential amino acids, 2 mM glutamine, and antibiotics: 100 IU/ml of penicillin and 100 μg/ml of streptomycin (all purchased from PAA Laboratories, Linz, Austria) under standard conditions at 37°C in an atmosphere of 95% air: 5% CO_2_. The cells were subcultured 1-2 times weekly.

### Chemicals

ATRA (Sigma Chemical Co., St. Louis, MO, USA) was prepared as a stock solution at the concentration of 100 mM in dimethyl sulfoxide (DMSO; Sigma). CA (Sigma) and CX (LKT Laboratories, Inc., St. Paul, MN, USA) were dissolved in DMSO at the concentrations of 130 and 100 mM, respectively. Reagents were stored at -20°C under light-free conditions.

### Induction of cell differentiation

Stock solutions were diluted in fresh cell culture medium to obtain final concentrations of 1 and 10 μM of ATRA, 13 and 52 μM of CA and 10 and 50 μM of CX. In all experiments, cells were seeded onto Petri dishes 24 h before the treatment, and untreated cells were used as a control. The experimental design was the same as in our previous study [17]: cell populations were treated with ATRA alone or with ATRA and inhibitor (CA or CX) in respective concentrations. However, a combined treatment with 10 μM ATRA and 50 μM CX was not included in these experiments due to the predominant cytotoxic effect on cell populations. Cells were harvested after three days of cultivation in the presence of ATRA and inhibitors.

### Expression profiling

Total RNA of treated cell populations was isolated using the GenElute™ Mammalian Total RNA Miniprep Kit (Sigma), and its concentration and integrity were determined spectrophotometrically. Conversion of experimental RNA to target cDNA and further amplification and biotin-UTP labeling was performed using TrueLabeling-AMP™ 2.0 cRNA (SABiosciences, Frederick, MD, USA). After purification of labeled target cRNA with the SuperArray ArrayGrade cRNA Cleanup Kit, the cRNA was hybridized to Human Cancer OHS-802 Oligo GEArray membranes that profile 440 genes (both SABiosciences). The expression levels of each gene were detected with chemiluminescence using the alkaline phosphatase-conjugated streptavidin substrate, and membranes were recorded using the MultiImage™ II Light Cabinet (DE-500) (Alpha Innotech Corp., CA, USA).

### Data processing and analysis

The image data were processed and analyzed by the GEArray Expression Analysis Suite software (SABiosciences) with background subtraction. All data were standardized as a ratio of gene expression intensity to the mean expression intensity of selected housekeeping genes (*ACTB, RPS27A, HSP90AB1*). Cluster analyses were performed using the GEArray Expression Analysis Suite software according to the design of the experiments, i.e., separately for each cell line and inhibitor type.

## Results

Our experiments were aimed at a detailed analysis of the changes in gene expression in SK-N-BE(2) and SH-SY5Y cells induced by combined treatment with ATRA and LOX/COX inhibitors (CA or CX). We used the same experimental design as in our previous study [17] that reported at the cellular level the influence of this treatment on cell differentiation and apoptosis: we evaluate cell populations treated with ATRA alone or with ATRA and inhibitor (CA or CX) in respective concentrations.

**Figure 1:**
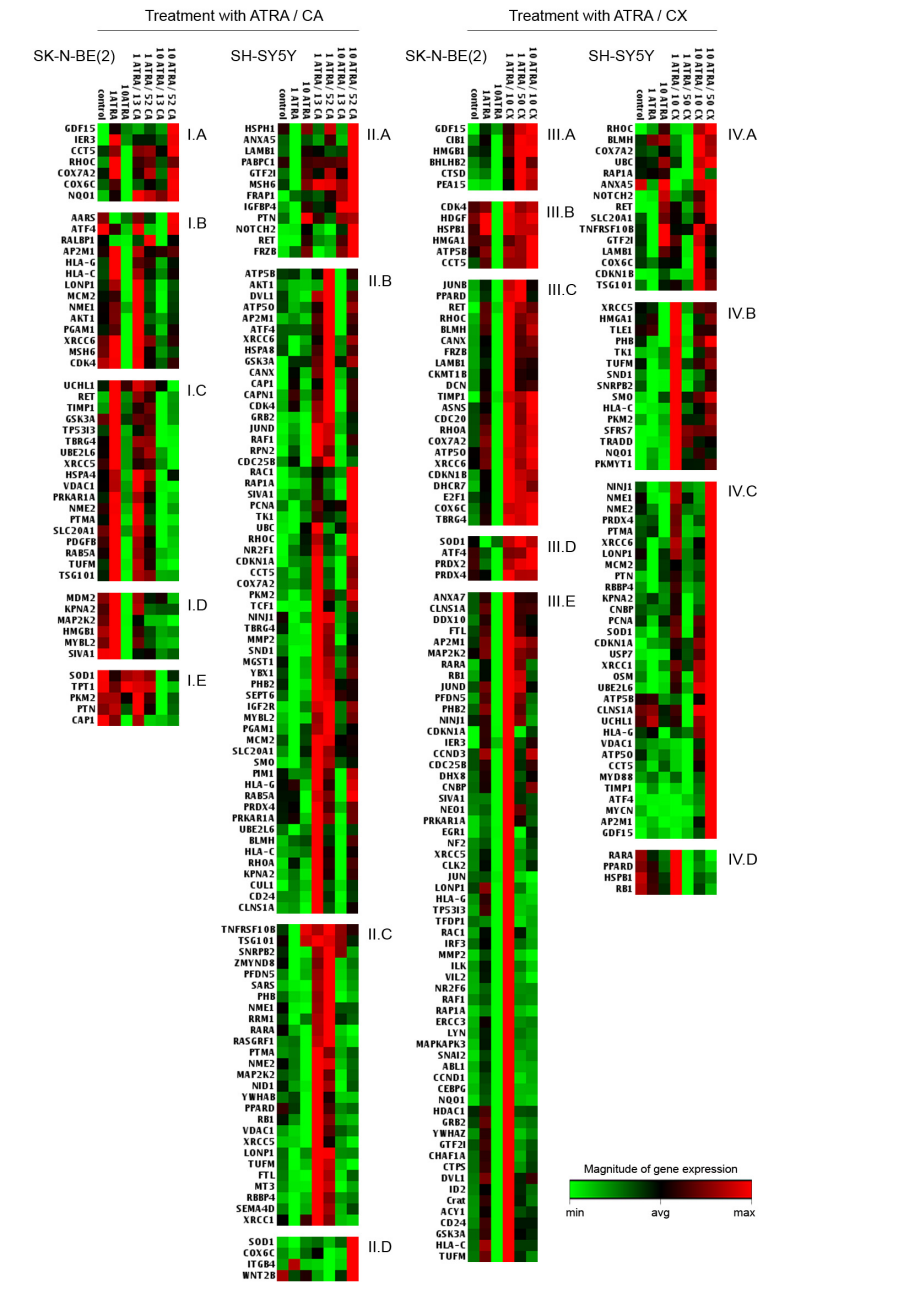
**Results of gene cluster analysis**. Genes were clustered according to type of changes in expression in particular cell lines (SK-N-BE(2) or SH-SY5Y) after combined treatment with ATRA and particular inhibitors (CA or CX). ATRA was applied in concentrations of 1 or 10 μM (1 ATRA, 10 ATRA); CA in concentrations of 13 and 52 μM (13 CA, 52 CA), and CX in concentrations of 10 and 50 μM (10 CX, 50 CX). The green color at the farthest left end of the color scale corresponds to the minimal value; the red color at the farthest right end of the color scale corresponds to the maximal value; and the black color in the middle of the color scale corresponds to the average value. Each of the other values corresponds to a certain color according to its magnitude. The colors are assigned according to the value of the particular gene expression in all samples in the respective experimental variant (I, II, III or IV).

We performed the comparison of cluster analyses of achieved data to detect genes or gene groups with the same types of changes in their expression (Figure 1, Table 1). After combined treatment with ATRA and CA, we detected 50 genes with changed expression in SK-N-BE(2) cells and 91 genes with changed expression in SH-SY5Y cells. As a result of combined treatment with ATRA and CX, 98 genes with changed expression were identified in SK-N-BE(2) cells and 66 genes with changed expression were identified in SH-SY5Y cells. We analyzed these data from two different viewpoints.

**Table 1:** Description of different types of changes in gene expression after combined treatment with ATRA and inhibitors (CA or CX) in SK-N-BE(2) and SH-SY5Y cell lines

cluster	number of genes	type of change in gene expression
**I. Treatment with ATRA and CA; SK-N-BE(2) cell line**		

I.A	7	strong increase especially after treatment with 10 ATRA/52 CA; marked increase noted also after treatment with 1 ATRA alone and all other combinations

I.B	14	marked increase especially after treatment with 1 ATRA/13 CA; the increase noted also after treatment with 1 ATRA alone

I.C	18	marked increase especially after treatment with 1 ATRA and also both combinations of ATRA with CA; 10 ATRA alone or in combinations with CA decreases gene expression

I.D	6	slight increase after treatment with 1 RA; marked decrease after treatment with 10 RA and all combinations

I.E	5	decrease after treatment with 10 ATRA in both combinations with CA

**II. Treatment with ATRA and CA; SH-SY5Y cell line**		

II.A	12	strong increase especially after treatment with 10 ATRA/52 CA; marked increase noted also after treatment with 10 ATRA alone and all other combinations in concentration-dependent manner

II.B	58	marked increase especially after treatment with 1 ATRA in both combinations with CA and also after treatment with 10 ATRA/52 CA; application of ATRA alone showed no influence on gene expression

II.C	27	marked increase after treatment with 1 ATRA in both combinations; application of ATRA alone and 10 ATRA in both combinations showed no influence on gene expression

II.D	4	strong increase after treatment with 10 ATRA/52 CA; application of ATRA alone and all other combinations showed no or minimal influence on gene expression

**III. Treatment with ATRA and CX; SK-N-BE(2) cell line**		

III.A	6	strong increase after treatment with 10 ATRA/10 CX and 1 ATRA/50 CX; slight increase after treatment with 1 ATRA/10 CX; application of ATRA alone showed no or minimal influence on gene expression

III.B	6	marked increase after treatment with ATRA in all combinations with CX; treatment with 1 ATRA alone showed the same effect on gene expression as observable in control cells

III.C	22	strong increase after treatment with ATRA in all combinations with CX; slight increase after treatment with 1 ATRA alone

III.D	4	marked increase after treatment with ATRA in all combinations with CX; decrease after treatment with ATRA alone

III.E	60	strong increase after treatment with 1 ATRA/10 CX; slight increase after treatment with 1 ATRA alone

**IV. Treatment with ATRA and CX; SH-SY5Y cell line**		

IV.A	15	marked increase after treatment with 10 ATRA alone and also in both combinations with CX; application of 1 ATRA alone or in combinations with CX showed no or minimal influence on gene expression

IV.B	15	strong increase after treatment with 1 ATRA/10 CX; slight increase after treatment with 10 ATRA in both combinations with CX

IV.C	32	strong increase after treatment with 10 ATRA/50 CX

IV.D	4	marked increase after treatment with 1 ATRA/10 CX; marked decrease after treatment with all other combinations

ATRA was applied in concentrations of 1 or 10 μM (1 ATRA, 10 ATRA); CA in concentrations of 13 and 52 μM (13 CA, 52 CA), and CX in concentrations of 10 and 50 μM (10 CX, 50 CX). All decriptions are related to the gene expression identified in control untreated cells.

First, we determined genes the expression of which was changed in the same cell line (SK-N-BE(2) or SH-SY5Y) after combined treatment with both ATRA and CA, or ATRA and CX. Under this criterion, we ascertained 25 genes in SK-N-BE(2) cells and 46 genes in SH-SY5Y cells (Table 2).

**Table 2:** Genes with changed expression detected in particular cell line (SK-N-BE(2) or SH-SY5Y) after combined treatment with ATRA and both inhibitors (CA or CX)

SK-N-BE(2) cell line	*AP2M1*, *ATF4*, *CCT5*, *CDK4, COX6C*, *COX7A2*, *GDF15, GSK3A, HLA-C*, *HLA-G*, *HMGB1, IER3, LONP1*, *MAP2K2, NQO1, PRKAR1A, RET*, *RHOC*, *SIVA1, SOD1, TBRG4, TIMP1, TP53I3, TUFM*, *XRCC5*, *XRCC6*
**SH-SY5Y cell line**	*ANXA5, AP2M1*, *ATF4*, *ATP5B, ATP5O, BLMH, CCT5*, *CDKN1A, CLNS1A, COX6C*, *COX7A2*, *GTF2I, HLA-C*, *HLA-G*, *KPNA2, LAMB1, LONP1*, *MCM2, NINJ1, NME1, NME2, PHB, PKM2, PPARD, PRDX4, PTMA, RAP1A, RARA, RB1, RBBP4, RET*, *RHOC*, *SLC20A1, SMO, SND1, SNRPB2, TK1, TNFRST10B, TSG101, TUFM*, *UBC, UBE2L6, VDAC1, XRCC1, XRCC5, XRCC6*

ATRA was applied in concentrations of 1 or 10 μM (1 ATRA, 10 ATRA); CA in concentrations of 13 and 52 μM (13 CA, 52 CA), and CX in concentrations of 10 and 50 μM (10 CX, 50 CX). The same genes influenced in both cell lines are underlined.

Second, we detected genes the expression of which was changed after treatment with the same inhibitor (CA or CX) in combination with ATRA in both cell lines. In this condition, we identified 37 genes with changed expression after combined treatment with ATRA and CA in both cell lines and 30 genes with changed expression after combined treatment with ATRA and CX in both cell lines (Table 3).

**Table 3:** Genes with changed expression detected after the same combined treatment (ATRA with CA or ATRA with CX) in both cell lines (SK-N-BE(2) and SH-SY5Y)

Treatment with ATRA and CA	*AKT1, AP2M1*, *ATF4*, *CAP1, CCT5*, *CDK4, CLNS1A*, *COX6C*, *COX7A2*, *GSK3A, HLA-C*, *HLA-G*, *KPNA2, LONP1*, *MAP2K2, MCM2, MSH6, MYBL2, NME1, NME2, PGAM1, PHB2, PKM2, PRKAR1A, PTMA, RAB5A, RET*, *RHOC*, *SIVA1, SLC20A1, TBRG4, TSG101, TUFM*, *UBE2L6, VDAC1, XRCC5*, *XRCC6*
**Treatment with ATRA and CX**	*AP2M1*, *ATF4*, *ATP5O, BLMH, CCT5*, *CDKN1A, CDKN1B, CLNS1A*, *COX6C*, *COX7A2*, *GDF15, HLA-C*, *HLA-G*, *HMGA1, HSPB1, LAMB1, LONP1*, *NQO1, PPARD, PRDX4, RAP1A, RARA, RB1, RET*, *RHOC*, *SOD1, TIMP1, TUFM*, *XRCC5*, *XRCC6*

ATRA was applied in concentrations of 1 or 10 μM (1 ATRA, 10 ATRA); CA in concentrations of 13 and 52 μM (13 CA, 52 CA), and CX in concentrations of 10 and 50 μM (10 CX, 50 CX). The same genes influenced by combinations with both inhibitors are underlined.

Most interestingly, we identified 14 genes with changed gene expression in both cell lines and after both combined treatments with ATRA and LOX/COX inhibitors (Tables 2, 3).

## Discussion

Retinoic acid and its derivatives are known to induce differentiation in leukemias as well as in several types of solid tumors, including neuroblastoma [1-3,13,18]. In our previous study, we reported the possibility of modulating the differentiation potential of ATRA in SK-N-BE(2) and SH-SY5Y neuroblastoma cell lines by combined treatment with ATRA and LOX/COX inhibitors, especially with CA as the inhibitor of 5-LOX [17]. The aim of this work was to investigate in detail the changes in gene expression of cancer-related genes in neuroblastoma cells after the same combined treatment as described in the previous study, with special regard to the genes involved in cell differentiation.

Based on the analysis of the expression of 440 cancer-related genes by the Human Cancer Oligo GEArray microarray, we noted an overall increase in gene expression and only a minimal number of downregulated genes after treatment with ATRA alone or with ATRA in combinations with CA or CX. These findings are not surprising with regard to known mechanisms of retinoid action: both RA and retinoids bind to the inducible nuclear retinoid receptors that function as transcriptional factors of genes with RA-responsive elements [18,19].

Our results in both cell lines clearly show the crucial role of the *RET *proto-oncogene in retinoid-induced cell differentiation in neuroblastoma cells. *RET *is overexpressed in both cell lines after the application of ATRA alone or in combination with CA or CX. However, these cell lines differ in their response sensitivity: *RET *expression is upregulated in SK-N-BE(2) by treatment with 1 μM ATRA and its combinations with CA or CX, whereas 10 μM ATRA (alone or in combination) is needed for the overexpression of *RET *in SH-SY5Y cells. These findings are completely in accordance both with other experiments on *RET *overexpression after retinoid-induced cell differentiation in the same neuroblastoma cell lines [18] and with our previous results with regard to the difference in response sensitivity [17]. Moreover, RET overexpression is associated with neuronal differentiation and correlates with the expression of NF-200 [17,20].

The other gene that is overexpressed in both cell lines after the application of ATRA alone or in combination with CA or CX is *RHOC*, which encodes a member of the Rho GTPase family. Proteins of this family, especially RhoA, Rac1 and Cdc24, are known to play an important role in actin cytoskeleton remodeling, and they are also involved in the neurite outgrowth and remodeling during neuronal differentiation [21,22]. Besides playing a role in the metastasis of some human cancers, namely of breast carcinomas [23], overexpression of the RhoC protein was detected in glial precursors during differentiation of fetal neuroepithelial cells [24]. The detected overexpression of *RHOC *in both cell lines after treatment, especially in a concentration-dependent manner after combined treatment with CX, suggests the possible participation of this molecule in retinoid-induced differentiation. In contrast, same changes in the expression of *RHOA *were observed only in SK-N-BE(2) cells treated with ATRA and CX. In SH-SY5Y cells, *RHOA *was overexpressed after treatment with 1 μM ATRA and especially with its combinations with CA, whereas the same effect for *RHOC *was detected after treatment with 10 μM ATRA in SK-N-BE(2) cells. These data are in accordance with the hypothesis that the expression and activity of RhoA, B, and C proteins in cancer cells may be altered in different ways [25].

Remodeling of the cytoskeleton seems to be an important part of the induced cell differentiation of neuroblastoma cells because cell morphology undergoes substantial changes during this process. The product of the *CTT5 *gene, i.e., chaperonin containing TCP-1, subunit epsilon, is generally involved in protein folding and assembly in the cytoplasm of eukaryotic cells [26], and it was reported as active in cytoskeleton rearrangements during neuritogenesis in mouse neuroblastoma cells, especially in the perikaryal region of the cytoplasm [27]. Because *CCT5 *is overexpressed in both cell lines after combined treatment with CA as well as with CX in a concentration-dependent manner, we can suppose that the protein participates in rearrangements of cytoskeletal components during induced neuronal differentiation. A similar function, i.e., participation in cytoskeleton rearrangements, was also reported in the case of the Tu translation elongation factor, a product of the *TUFM *gene [28], which was detected as overexpressed in both cell lines after combined treatment with CA as well as with CX.

Taken together, overexpression of the genes listed above was detected in our experiments as a common phenomenon in both cell lines as a result of combined treatment with ATRA and inhibitor (CA or CX). Overexpression of the *RET *protooncogene is generally associated with retinoid-induced cell differentiation. Products of other genes, i.e., *RHOC*, *RHOA*, *CCT5 *and *TUFM*, were reported as also being involved in cytoskeleton rearrangements that are necessary for changes of cell morphology during the neuronal differentiation of neuroblastoma cells. The common overexpression of these genes in both cell lines independent of the inhibitor used (CA or CX) and mostly in a concentration-dependent manner suggests that they participate in the process of cell differentiation induced by ATRA and potentiated by both CA and CX. This hypothesis is supported by the observation of initial changes in cell morphology in both cell lines at day two after treatment in the same experimental design [17].

Moreover, our previous study suggested a higher sensitivity of SK-N-BE(2) cells to the induced differentiation, especially by combined treatment with ATRA and CA (17). In this cell line, we found strong overexpression of the *GDF15 *gene after combined treatment with ATRA and inhibitor (CA or CX) in a concentration-dependent manner. Overexpression of *GDF15 *(also known as *MIC-1*, *NAG-1, PDF*, *PLAB*, or *PTGFB*) was reported as a result of the induced neuronal differentiation of PC12 cells [29]. Despite various effects of this cytokine, as described in many types of human cancer cells, its proapoptotic and antitumorigenic role is widely accepted, and an increase in its expression by COX-inhibitors has been proved [30]. In contrast, other authors suggest that the activity of this cytokine is not related to the COX-2 expression and that it seems to be cell type-specific [31]. An increase in the expression of the *IER3 *encoding transcriptional factor, immediate early response 3, was reported in the SK-N-BE(2)C neuroblastoma cell line during retinoic acid-induced neuronal differentiation [32]; our findings in the SK-N-BE(2) cell line are completely in accordance with these results.

However, overexpression of *NME1 *and *NME2 *genes was found only in SH-SY5Y cells after combined treatment with ATRA and inhibitors. The overexpression of this gene family was reported to be associated with more differentiated phenotypes in human and murine neuroblastoma cell lines [33-35]. Similar changes were observed in the SH-SY5Y cell line and in the expression of the *CDKN1A *gene after combined treatment with ATRA and both inhibitors; the *CDKN1B *gene was overexpressed in SH-SY5Y cells with a combination of ATRA and CX only. An increase in the expression of cyclin kinase inhibitors by RA alone and in combination with histone deacetylase inhibitors was reported [36]. Moreover, inhibition of cdk activity was repeatedly confirmed to be a determinant of neuronal differentiation [37]. The same expression pattern was found in SH-SY5Y cells and for the *NINJ1 *gene; this gene encodes adhesion molecules promoting neurite outgrowth [38]. RA-induced differentiation of neuroblastoma cells is also associated with the overexpression of tumor necrosis factor receptors (TNFRs) [39]. In SH-SY5Y cells, we noted an increase in the expression of the *TNFRST10B *gene after treatment both with 10 μM ATRA alone and with all combinations of ATRA and inhibitors.

To summarize, in addition to the genes generally overexpressed in both cell lines after combined treatment, as listed above, we also identified other genes that are specifically influenced in specific cell lines, including SK-N-BE(2) or SH-SY5Y. These genes are also known to be involved in the process of neuronal differentiation in neuroblastoma cells; however, their regulation is obviously cell type-specific and is independent of the inhibitor type.

Nevertheless, we also determined sets of genes influenced specifically by combined treatment with ATRA and CA in both SK-N-BE(2) and SH-SY5Y cell lines; but changes in the gene expression of such genes may differ between these cell lines. In contrast, the very same increase of *AKT1 *gene expression in both cell lines treated with the combination of 1 μM ATRA and CA was observed. Published results on SH-SY5Y cells suggest that the PI3K/Akt signaling pathway is activated during RA-induced differentiation [40].

We also identified genes influenced specifically by the combined treatment with ATRA and CX in both SK-N-BE(2) and SH-SY5Y cell lines. The most interesting finding is the overexpression of the *HMGA1 *gene in both cell lines after combined treatment with ATRA and CX in a concentration-dependent manner. According to published data, retinoic acid may increase *HMGA1 *expression in RA-resistant neuroblastoma cells, but it inhibits this expression in cells undergoing RA-induced neuronal differentiation [41]. Nevertheless, *HMGA1 *expression is influenced by *MYCN *status in neuroblastoma cells: this gene is significantly more expressed in *MYCN*-amplified neuroblastomas and it might be also activated by c-MYC or other transcription factors [42]. Fort this reason, a detailed investigation of the *HMGA1 *expression in neuroblastoma cell lines treated with ATRA and LOX/COX inhibitors is needed.

Metronomic chemotherapy refers to the prolonged administration of low-dose cytotoxic and/or anti-angiogenic agents. This approach was reported to be potentially effective in the treatment of relapsed and poor-prognosis pediatric cancers, even in neuroblastoma [15] and CNS tumors [43]. In both these reports, chemotherapy agents were combined with administration of celecoxibe and isotretinoin. In context of our previous results [17] and especially of these data on expression profiling, therapeutic usage of retinoid in combination with COX inhibitor has strong biological rationale. Moreover, dietary uptake of the natural phenolic compounds including caffeic acid, for example, in honey, apple juice, grapes and some vegetables may also potentiate the cell differentiation induced by retinoids [44-46]. For these reasons, phase I/II clinical trials are highly warranted to further testing of the promising effect of LOX/COX inhibitors on retinoid-induced differentiation in pediatric cancer patients.

## Conclusion

These data support our initial hypothesis that ATRA-induced cell differentiation may be modulated by the combined application with LOX/COX inhibitors. Using expression profiling, we identified common changes in the expression of genes involved especially in cytoskeleton rearrangements that accompany neuronal differentiation of neuroblastoma cells. Not surprisingly, we also noted nonspecific activation of genes involved in reparation processes or that participate in the cell response to oxidative stress (for example, *XRCC5*, *XRCC6*, *NQO1*, *SOD1*, etc.). Nevertheless, the detected increase in expression of genes related to cell differentiation, mostly in a concentration-dependent manner (both for ATRA and inhibitors), suggests that the ATRA-induced differentiation of neuroblastoma cells may be enhanced by compounds affecting the intracellular metabolism of ATRA, especially via inhibition of arachidonic acid metabolic pathway.

## List of abbreviations

ATRA: all-*trans *retinoic acid; CA: caffeic acid; CX: celecoxib; COX: cyclooxygenase; LOX: lipoxygenase.

## Competing interests

The authors declare that they have no competing interests.

## Authors' contributions

PC carried out the experiments with cell lines, performed expression profiling and drafted the manuscript. MR participated in the experiments with cell lines and in the manuscript preparation. KZ and MH participated in the result analysis and in the manuscript preparation. JS coordinated this study and participated in the manuscript preparation. RV conceived the study, participated in the result analysis and drafted the manuscript. All authors read and approved the final manuscript.
